# Effects of Cereal Byproducts on Antioxidant Capacity of Black Soldier Fly Oil

**DOI:** 10.1002/fsn3.72164

**Published:** 2026-07-31

**Authors:** Yung‐Sheng Lin, Yu‐Ting Zhu, Cheng‐Kun He, Cheng‐You Chen, Chin‐Tung Wu

**Affiliations:** ^1^ Department of Chemical Engineering National United University Miaoli Taiwan; ^2^ Graduate School of Intelligent Data Science National Yunlin University of Science &Technology Douliu Taiwan

## Abstract

Black soldier fly (BSF) larvae efficiently convert organic residues into protein‐ and lipid‐rich biomass, with oil extracted from BSF larvae exhibiting notable antioxidant potential. This study investigated larval growth, oil yield, and antioxidant properties for four feeding substrates made from cereal byproducts: wheat bran, rice bran, sorghum grain, and red quinoa hulls. BSF larvae reared for 7 days on different substrates displayed significant differences in body size, moisture content, and oil extraction efficiency. Wheat‐bran‐fed larvae had the lowest moisture content and largest size, whereas red‐quinoa‐fed larvae exhibited the highest moisture content but smallest size. Sorghum‐fed larvae had the highest oil yield, followed by larvae fed with wheat bran, red quinoa, or rice bran. Oils extracted from larvae reared on the four substrates were analyzed for total phenolic content (TPC) and antioxidant activity using the 2,2‐diphenyl‐1‐picrylhydrazyl (DPPH), 2,2′‐azinobis‐3‐ethylbenzothiazoline‐6‐sulfonic acid (ABTS), and ferric reducing antioxidant power (FRAP) assays. Oil extracted from red‐quinoa‐fed larvae had the highest TPC (79.25 ± 1.04 μg of gallic acid equivalents per gram of oil) and strongest radical scavenging abilities (IC_50_ for DPPH = 22.0 ± 1.1 μg/mL, IC_50_ for ABTS = 11.6 ± 1.0 μg/mL, and FRAP = 29.24 ± 1.00 μg of vitamin C equivalents per gram of oil), closely followed by oil extracted from rice‐bran‐fed larvae. Pearson correlations indicated that TPC strongly predicted antioxidant performance (*r* = −0.995 to 0.962, *p* < 0.01). Red quinoa optimizes the functional quality of BSF oil. Rice bran offers a balance between oil yield and bioactivity. These findings highlight the effects of feed substrate on BSF oil properties.

## Graphical abstract

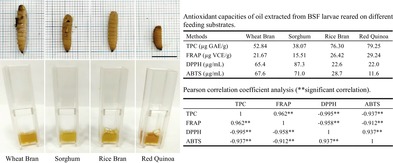

## 1 Introduction

Oxidative stress occurs when the production of reactive oxygen and nitrogen species exceeds the capacity of the body to defend against oxidative forces, resulting in damage to lipids, proteins, and nucleic acids (Husain and Kumar 2012; Persson et al. 2014). Under normal conditions, low levels of reactive oxygen and nitrogen species are involved in cellular signaling. Excessive accumulation of the aforementioned species results in the initiation of lipid peroxidation, enzyme inactivation, and DNA strand breaks and ultimately the impairment of cell integrity (Kurutas 2016; Pisoschi and Pop 2015). Because of the limited capacity of human endogenous antioxidant systems, dietary antioxidants are essential for maintaining redox balance and preventing disease (Halliwell and Gutteridge 2015). Dietary antioxidants include a broad array of compounds, such as polyphenols, flavonoids, carotenoids, and vitamins C and E. These compounds neutralize free radicals or chelate transition metals, thereby interrupting oxidative chain reactions (Neha et al. 2019). Epidemiological studies have linked high intake of antioxidant‐rich foods with reduced risk of cancer, cardiovascular disease, and photoaging‐related skin damage (Aruoma 1998; Meagher and Rader 2001; Rahman et al. 2012; Witztum and Berliner 1998; Yousri et al. 2011). In the context of skin health, ultraviolet‐irradiation‐induced formation of reactive species leads to sunburn, collagen breakdown, and inflammatory responses, all of which can be mitigated by antioxidant compounds (Darvin et al. 2008; Jeon et al. 2009; Masaki 2010).

Insect‐derived lipids have attracted attention as alternative bioactive ingredients with potential nutraceutical and cosmeceutical applications. The black soldier fly (BSF; *Hermetia illucens* ) has emerged as a promising species in this context because of its efficiency in bioconverting organic waste into biomass rich in proteins and lipids. BSF larvae produce oil that contains medium‐chain fatty acids, especially lauric acid, which has antimicrobial and antioxidant properties (Almeida et al. 2022). Studies have reported that the oil extracted from BSF larvae exhibits high antioxidant capacity, with its scavenging activity for 2,2‐diphenyl‐1‐picrylhydrazyl (DPPH) being up to 96.6%. Moreover, BSF oil ranks second to krill oil in terms of ferric reducing ability (Phongpradist et al. 2023). The total phenolic content (TPC) of insect oil is positively correlated with antioxidant capacity, indicating that phenolic compounds are key contributors to bioactivity (Kim et al. 2018, 2020; Mohammad Taghi Gharibzahedi and Altintas 2023; Muangrat and Pannasai 2024).

Beyond waste reduction, recent advances in circular economy principles have positioned the BSF as a tool for upcycling nutrients into high‐value functional lipids (Ameixa et al. 2023). Agricultural byproducts such as wheat bran, rice bran, sorghum grain, and red quinoa hulls are rich in phenolic acids, flavonoids, tocopherols, and other bioactive compounds (Papageorgiou and Skendi 2018). Compared with more extreme substrates such as fibrous palm kernel meal, highly heterogeneous kitchen waste, or protein‐dense brewery co‐products, cereal byproducts provide nutritionally intermediate and compositionally stable substrates, allowing substrate bioactivity relationships to be evaluated under controlled and reproducible conditions (Bajra et al. 2025; Shumo et al. 2019). While the bioconversion efficiency of BSF is well‐documented, the ability to modulate the bioactivity of insect oil through specific dietary interventions remains a critical research gap (IJdema et al. 2025). The use of these low‐cost residues as feeding substrates might enhance the functional quality of BSF oil while valorizing agricultural waste. Research has indicated that extraction solvent and feed composition influence the properties of insect oil (Almeida et al. 2022; Ravi et al. 2019). Almeida et al. (2022) found that hexane and acetone extraction resulted in the acquisition of oils with similar fatty acid profiles but different antioxidant activities, which varied with the oil concentration. Phongpradist et al. (2023) noted high activity in DPPH scavenging and ferric reduction assays. Furthermore, Kim et al. (2018), Mohammad Taghi Gharibzahedi and Altintas (2023), and Muangrat and Pannasai (2024) have found that higher phenolic content is associated with improved radical scavenging. Despite these advancements, no study has systematically compared how wheat bran, rice bran, sorghum grain, and red quinoa hulls influence the antioxidant properties and oil yield of BSF‐derived products. This comparative analysis is essential for understanding how substrate selection modulates oil bioactivity while optimizing production efficiency (Shumo et al. 2019; Siddiqui et al. 2025). Identifying optimal substrates would enable BSF farming operations to produce oils with targeted bioactive profiles for specific market applications, such as premium antioxidant supplements as opposed to commodity lipids, thereby supporting both agricultural waste valorization and functional ingredient development.

To address this gap, this study aims to systematically evaluate the yields and antioxidant profiles of BSF‐derived oil reared on these four distinctive cereal byproduct substrates. Specifically, BSF larvae were reared under controlled conditions on wheat bran, rice bran, sorghum grain, and red quinoa hulls, and the resulting insect oil was extracted via a hexane‐based organic solvent method. We systematically determined the TPC and evaluated the antioxidant profiles of the extracted BSF oil using DPPH, 2,2′‐azinobis‐3‐ethylbenzothiazoline‐6‐sulfonic acid (ABTS), and ferric reducing antioxidant power (FRAP) assays. Through correlation analysis, this study establishes the quantitative relationships between cereal substrate composition and BSF oil antioxidant capacity, providing evidence‐based substrate selection criteria for optimizing both oil yield and functional quality in commercial production.

## 2 Materials and Methods

### 2.1 Insect Treatment

The BSF larvae used in this study were 2 weeks old and were provided by Wormax, Taiwan. The larvae were divided into 12 groups, each of which weighed 100 g. Each group was placed into one of four preprepared feed treatments, with three replicates produced per feed type (i.e., each feed type was used to rear 100 g of larvae per replicate). The four preprepared feed treatments were based on cereal byproducts (wheat bran, sorghum, rice bran, and red quinoa). Before feeding, distilled water was added to each feed ingredient and thoroughly mixed to adjust the substrate moisture content to approximately 65% and to ensure a homogeneous substrate. For each replicate, 3000 g of the prepared substrate (wet weight) were provided as a single feeding at the beginning of the 7‐day rearing period, and no additional feed was supplied thereafter. The total rearing period lasted 7 days, at the end of which the larvae were separated from the feed by using a sieving machine. The separated BSF larvae were first subjected to blanching in boiling water for 10 min to ensure sterilization. Subsequently, they were drained and air‐dried for 1 day, at which point they were considered fresh larvae. The drying methods (oven‐drying at 65°C–70°C for 24–48 h) used in other relevant studies (Hurtado‐Ribeira, Hernández, et al. 2023; Hurtado‐Ribeira, Silvan, et al. 2023; Santos Filipe et al. 2024) were optimized, and the fresh larvae were placed in an oven at 80°C for 72 h to ensure complete dehydration. Finally, the dried larvae were ground into fine powder by using a milling machine and stored at −80°C for further analysis.

### 2.2 Insect Oil Extraction

This study adopted the organic solvent soaking method of Almeida et al. (2022) for insect oil extraction because of its high yield and simple operation (Zhang et al. 2018). Hexane was selected as the organic solvent because of its widespread use for lipid extraction (Muangrat and Pannasai 2024; Ravi et al. 2019; Yi et al. 2013) and its high extraction yield (Ayoola et al. 2014; Esther et al. 2023). In the extraction process, processed insect powder was placed in a 2 L plastic container, fully submerged in hexane, and then sealed for further analysis. The upper organic phase was then collected, and hexane was removed using a vacuum rotary evaporator at 45°C to extract oil. This procedure was repeated multiple times to maximize extraction efficiency. After extraction, the oil was weighed, and its yield (%) was determined as follows:

$$\begin{array}{c} \text{Oil extraction yield}\;\left(\%\right)=\\ \left(\text{total weight of oil}/\text{total weight of larval powder}\right)\times 100\%. \end{array}$$

The moisture content of the larvae was determined by measuring the difference between their fresh and dry weights. Freshly harvested larvae were weighed and then dried in an oven at a constant temperature until their weight stabilized. The moisture content was then calculated using the following equation:

$$\begin{array}{c} \text{Moisture content}\;\left(\%\right)=\\ \left[\left(\text{fresh weight}-\mathrm{dry}\;\text{weight}\right)/\text{fresh weight}\right]\times 100\%. \end{array}$$

### 2.3 Determination of TPC

This study modified the experimental methods used in other research (Chen et al. 2021; Lin et al. 2024; Wu et al. 2021) to determine TPC. A 0.35 g oil sample was diluted to 1000 μL by using 95% ethanol. The sample was then vortexed and centrifuged. Subsequently, 200 μL of the upper layer of the diluted solution was mixed with 200 μL of 0.3 M Folin–Ciocalteu phenol reagent and vortexed for 15 s. Next, 200 μL of 10% sodium carbonate solution and 400 μL of ultrapure (deionized) water were added to the aforementioned solution. The mixture was left in the dark for 1 h before being centrifuged at 5000 rpm for 15 min. After centrifugation, 100 μL of the supernatant was transferred to a microplate and analyzed using a full‐spectrum optical spectrophotometer to determine the sample's 700 nm absorbance. The aforementioned procedure was performed thrice.

To determine TPC, a calibration curve of gallic acid (concentration range of 20–130 μg/mL) was used. The results were obtained in micrograms of gallic acid equivalents (GAEs) per gram of oil (μg GAEs/g oil).

### 2.4 FRAP Assay

This study adapted experimental methods from other studies (Chen et al. 2021; Wu et al. 2021) to investigate the ferric reducing power of the extracted oil. A 0.525 g oil sample was diluted to 1000 μL by using 95% ethanol. The sample was then vortexed and centrifuged. Subsequently, 200 μL of the upper layer of the diluted solution was mixed with 100 μL of 1% K_3_Fe(CN)_6_ and 100 μL of 0.2 M phosphate‐buffered saline. The mixture was heated in a 50°C water bath for 20 min and then rapidly cooled for 3 min. Next, 100 μL of 10% trichloroacetic acid was added to the mixture, following which centrifugation was conducted at 3000 rpm for 10 min. After centrifugation, 100 μL of the supernatant was mixed with 25 μL of 0.1% FeCl_3_ and 100 μL of ultrapure (deionized) water. The mixture was left to react in the dark for 10 min. Subsequently, 100 μL of the supernatant was transferred to a microplate and analyzed using a full‐spectrum optical spectrophotometer to determine the sample's 700‐nm absorbance. Each sample was tested in triplicate. A calibration curve was established using vitamin C solutions with a concentration of 10–100 μg/mL. The results were obtained in micrograms of vitamin C equivalents (VCEs) per gram of oil (μg VCEs/g oil).

### 2.5 DPPH Scavenging Activity

This study modified methods from other research (Chen et al. 2021; Lin et al. 2024; Wu et al. 2021) to examine the DPPH scavenging activity of the extracted oil. An appropriate quantity of oil was mixed with 95% ethanol solution, vortexed for 15 s, and then centrifuged for 10 min at 5000 rpm. Next, the upper layer (200 μL) of the solution was mixed with 200 μL of 200 μM DPPH ethanol solution and allowed to react in the dark for 30 min. Subsequently, 300 μL of the clarified solution was transferred to a microplate and analyzed using a full‐spectrum optical spectrophotometer to determine the sample's 517 nm absorbance. The aforementioned process was performed thrice. Vitamin C was used as the standard, and a calibration curve was established using VCE solutions with a concentration of 3–10 μg/mL. The DPPH scavenging activity was determined in terms of IC_50_ (μg/mL), which represents the sample concentration required to scavenge 50% of free radicals. A smaller IC_50_ value indicates better scavenging ability.

### 2.6 ABTS Scavenging Activity

This study adapted methods used in other studies (Chen et al. 2021; Lin et al. 2024; Wu et al. 2021) to determine the ABTS scavenging activity of the extracted oil. An appropriate quantity of oil sample was mixed with 95% ethanol solution, vortexed for 15 s, and then centrifuged for 10 min at 5000 rpm. Next, the upper layer (200 μL) of the solution was mixed with 200 μL of a precalibrated ABTS solution (absorbance: 0.7 ± 0.05), and reaction was allowed to occur in the dark for 30 min. Subsequently, 300 μL of the clarified solution was transferred to a microplate and analyzed using a full‐spectrum optical spectrophotometer to determine the sample's 517 nm absorbance. The aforementioned process was performed thrice. Trolox was used as the standard, and a calibration curve was established using Trolox solutions with a concentration of 3–10 μg/mL. The ABTS scavenging activity was also determined in terms of IC_50_ (μg/mL).

### 2.7 Statistical Analysis

This study used SPSS Statistics version 12.0 for statistical analysis. One‐way analysis of variance (ANOVA) was conducted to analyze the differences between different extracts. Post hoc analyses were performed using the Scheffé method. Finally, the Pearson product–moment correlation coefficient was used to assess the correlation between TPC and antioxidant activity. Statistical significance was assessed at *α* = 0.05 for pairwise comparisons and *α* = 0.01 for correlation analyses (Pearson coefficient).

## 3 Results and Discussion

### 3.1 Morphology and Extraction Yield

**FIGURE 1:**
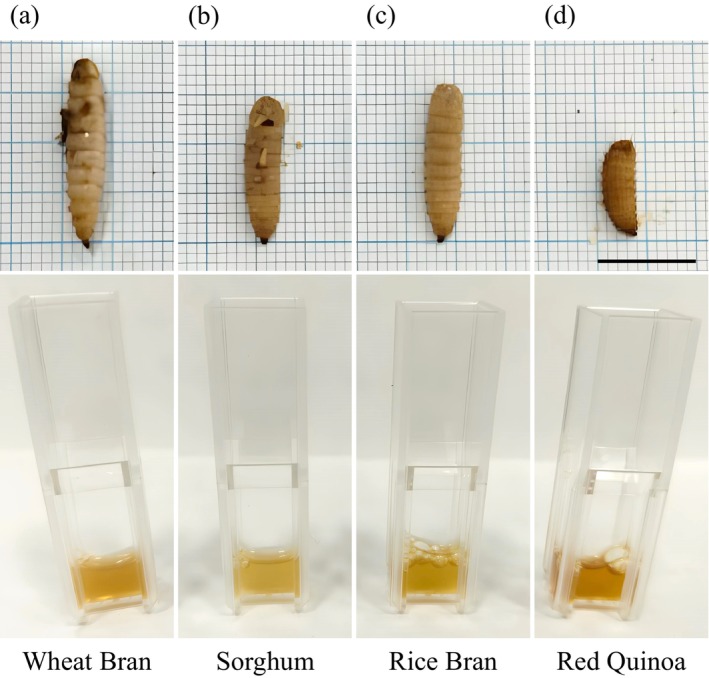
Growth morphologies of BSF larvae reared for 7 days on different feed substrates and corresponding larval oil colors: (a) Wheat bran, (b) Sorghum, (c) Rice bran, (d) Red quinoa. Scale bar: 1 cm.

The larvae reared on wheat bran exhibited a significantly larger body size on day 7 than did those reared on the other substrates (Figure 1). Larvae fed with sorghum and rice bran were moderately sized and did not show a significant size difference. The smallest larvae were those fed with red quinoa. As presented in Table 1, the larvae fed with red quinoa had the highest body moisture content (82.4% ± 0.2%), followed by those fed with sorghum (75.1% ± 0.9%), rice bran (73.1% ± 0.8%), and wheat bran (69.5% ± 0.7%). The type of feed also influenced lipid accumulation and oil extraction efficiency. Larvae fed with sorghum exhibited the highest oil extraction rate (23.96%), followed by those fed with wheat bran (22.61%), red quinoa (20.27%), and rice bran (17.42%). These results suggest that sorghum and wheat bran are more effective than rice bran and red quinoa in promoting lipid accumulation in BSF larvae, with rice bran showing the least effectiveness among the four feeding substrates. In addition, visible differences were noted in the colors of the larval oils extracted from different feeding substrates. As displayed in Figure 1, the oil produced by larvae fed with red quinoa exhibited a slightly reddish‐orange hue, whereas the oils produced by the other larvae had similar yellow‐brown appearances.

**TABLE 1:** Moisture content and oil extraction yield of BSF larvae reared on different feed substrates.

	Wheat Bran	Sorghum	Rice Bran	Red Quinoa
Moisture content (%)	69.5 ± 0.7	75.1 ± 0.9	73.1 ± 0.8	82.4 ± 0.2
Oil extraction yield (%)	22.61	23.96	17.42	20.27

### 3.2 Total Phenolic Content

**FIGURE 2:**
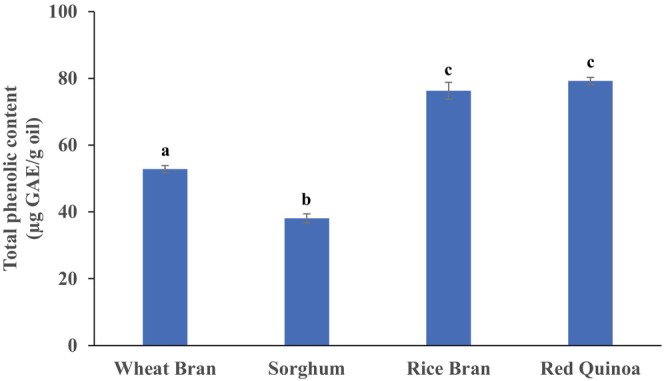
TPC of oils extracted from BSF larvae reared on different feeding substrates. Different letters denote significant differences (one‐way ANOVA, *F* = 439.35, *p* < 0.01).

TPC of each oil produced by BSF larvae reared on the four feeding substrates was quantified by using gallic acid as the calibration standard. As shown in Figure 2, the oil extracted from larvae reared on red quinoa exhibited the highest TPC (79.25 ± 1.04 μg GAE/g oil), followed by the oils extracted from larvae fed reared on rice bran (76.30 ± 2.55 μg GAE/g oil), wheat bran (52.84 ± 1.07 μg GAE/g oil), and sorghum (38.07 ± 1.35 μg GAE/g oil). TPC of oil did not significantly differ between that extracted from red‐quinoa‐fed and rice‐bran‐fed larvae (*p* > 0.05); TPC of oil from these larvae was significantly higher than that of oil extracted from larvae reared on wheat bran or sorghum (*p* < 0.05). TPC of oil extracted from sorghum‐fed larvae was significantly lower than that of oil extracted from larvae reared on the other feeding substrates (*p* < 0.05). This TPC was less than half of that of oil extracted from larvae reared on red quinoa or rice bran. Nevertheless, phenolic compounds were detected in every oil sample, indicating that the oil of BSF larvae has intrinsic antioxidant potential, with the most pronounced antioxidant effects exhibited by oils extracted from larvae reared on red quinoa or rice bran substrates.

### 3.3 Ferric Reducing Antioxidant Power

**FIGURE 3:**
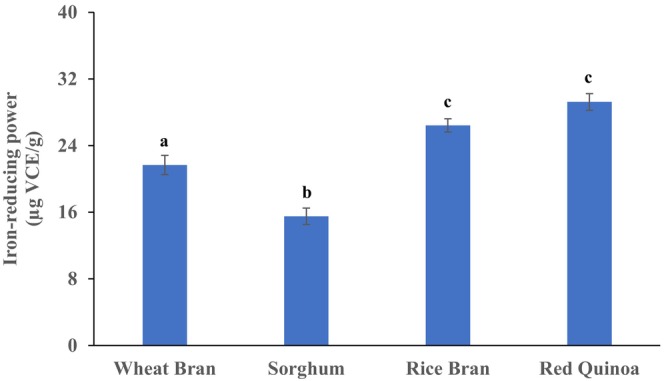
Ferric reducing power of oils extracted from BSF larvae reared on different feeding substrates. Different letters denote significant differences (one‐way ANOVA, *F* = 109.42, *p* < 0.01).

Reducing power refers to an antioxidant's ability to donate electrons and thereby inhibit oxidative reactions; thus, electron donation is a principal antioxidation mechanism (Zengin et al. 2015). In this study, the FRAP assay was used to evaluate the ferric reducing capacity of oils extracted from larvae reared on the four feeding substrates. As depicted in Figure 3, oil extracted from red‐quinoa‐fed larvae exhibited the highest reducing power (29.24 ± 1.00 μg VCE/g oil), followed by oil extracted from larvae reared on rice bran (26.42 ± 0.80 μg VCE/g oil), wheat bran (21.67 ± 1.15 μg VCE/g oil), or sorghum (15.51 ± 0.99 μg VCE/g oil). The reducing powers of the oils extracted from larvae reared on red quinoa or rice bran oil were not significantly different (difference of approximately 3 μg VCE/g oil, *p* > 0.05); however, the reducing powers of these oils were significantly different from those extracted from larvae reared on wheat bran or sorghum (*p* < 0.05). The reducing power of the oil extracted from sorghum‐fed larvae was significantly lower than those of the oils extracted from the larvae reared on other feeding substrates (*p* < 0.05).

### 3.4 DPPH Activity

**FIGURE 4:**
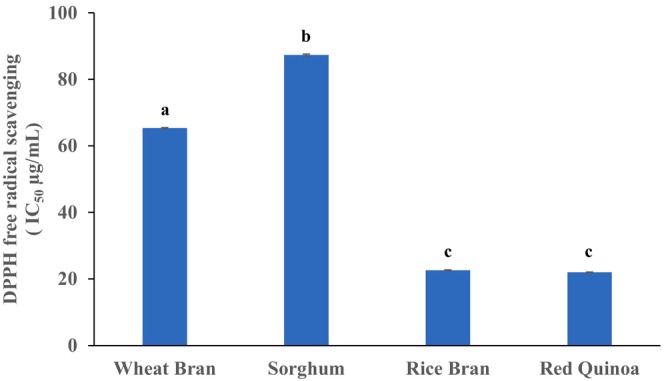
DPPH scavenging activities of oils extracted from BSF larvae reared on different feeding substrates. Different letters denote significant differences (one‐way ANOVA, *F* = 957.91, *p* < 0.01).

DPPH is a stable free radical that appears deep purple when dissolved in ethanol. When antioxidants scavenge DPPH radicals, the solution color changes from deep purple to light yellow. This study evaluated the DPPH scavenging activity of the produced oils. As shown in Figure 4, the oil extracted from red‐quinoa‐fed larvae exhibited the lowest IC_50_ value for DPPH (22.0 ± 1.1 μg/mL), followed by the oils extracted from larvae reared on rice bran (IC_50_ = 22.6 ± 1.2 μg/mL), wheat bran (IC_50_ = 65.4 ± 0.9 μg/mL), and sorghum (IC_50_ = 87.3 ± 3.1 μg/mL). The IC_50_ values of the oils extracted from larvae reared on red quinoa and rice bran were not significantly different from each other (*p* > 0.05) but were significantly lower than those of the oils extracted from larvae reared on wheat bran or sorghum (*p* < 0.05). The oil extracted from sorghum‐fed larvae exhibited a significantly higher IC_50_ value than did all other groups (*p* < 0.05). Overall, all oils exhibited antioxidant activity in the DPPH assay. The antioxidant activities of oils extracted from larvae reared on red quinoa or rice bran were approximately 3–4 fold higher than those of the oils extracted from larvae reared on wheat bran or sorghum.

### 3.5 ABTS Scavenging Activity

**FIGURE 5:**
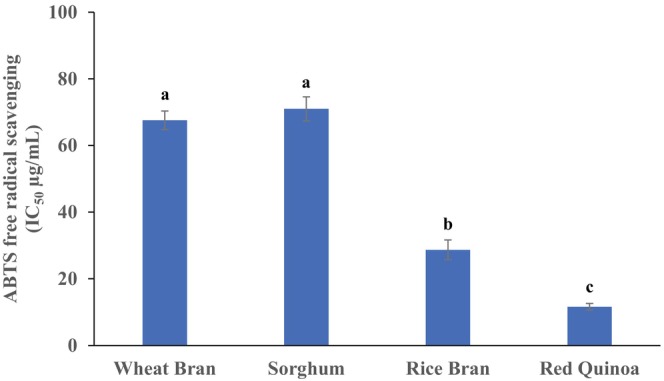
ABTS scavenging activities of oils extracted from BSF larvae reared on different feeding substrates. Different letters denote significant differences (One‐way ANOVA, *F* = 334.29, *p* < 0.01).

ABTS•^+^ is a bluish‐green cation, and its reduction by antioxidants results in solution decolorization. This study assessed the ABTS scavenging activities of the produced oils. As displayed in Figure 5, the oil extracted from red‐quinoa‐fed larvae exhibited a significantly lower (*p* < 0.05) IC_50_ value for ABTS (11.6 ±1.0 μg/mL) than did the oils extracted from larvae reared on rice bran (28.7 ± 3.0 μg/mL), wheat bran (67.6 ± 2.8 μg/mL), or sorghum (71.0 ± 3.6 μg/mL). The IC_50_ values of the oils extracted from the larvae reared on wheat bran or sorghum were not significantly different from each other (*p* > 0.05) but were significantly higher than those of the oils extracted from larvae reared on red quinoa or rice bran (*p* < 0.05). All four oils exhibited ABTS scavenging ability, with the oil extracted from red‐quinoa‐fed larvae exhibiting the highest ABTS scavenging activity (lowest IC_50_ value). The oils extracted from larvae reared on sorghum or wheat bran exhibited the lowest ABTS scavenging activities, and the oil extracted from rice‐bran‐fed larvae exhibited intermediate ABTS scavenging activity.

### 3.6 Pearson Product–Moment Correlation Coefficient

The Pearson product–moment correlation coefficient (*r*) ranges between 1 and −1, with values closer to 1 and −1 indicating stronger positive and negative correlations, respectively. The correlation is considered significant when *p* < 0.01. Table 2 presents the results of all antioxidant activity tests conducted in this study. These results indicated that TPC had significant correlations with antioxidant activities, as measured using the FRAP, DPPH, and ABTS assays (Table 3), with the corresponding Pearson correlation coefficients ranging from −0.995 to 0.962. TPC exhibited the strongest correlation with DPPH scavenging activity (*r* = −0.995, *p* < 0.01), followed by FRAP (*r* = 0.962, *p* < 0.01) and ABTS scavenging activity (*r* = −0.937, *p* < 0.01). These results indicate that higher TPC is associated with stronger antioxidant activity. Moreover, significant correlations were found between FRAP and DPPH scavenging activities (*r* = −0.958, *p* < 0.01), between FRAP and ABTS scavenging activities (*r* = −0.912, *p* < 0.01), and between DPPH and ABTS scavenging activities (*r* = 0.937, *p* < 0.01). These results suggest that all four adopted methods are interrelated, with an increase in one parameter likely accompanied by an increase in the others.

**TABLE 2:** Antioxidant capacities of oil extracted from BSF larvae reared on different feeding substrates.

Methods	Wheat Bran	Sorghum	Rice Bran	Red Quinoa
TPC (μg GAE/g)	52.84	38.07	76.30	79.25
FRAP (μg VCE/g)	21.67	15.51	26.42	29.24
DPPH (μg/mL)	65.4	87.3	22.6	22.0
ABTS (μg/mL)	67.6	71.0	28.7	11.6

**TABLE 3:** Pearson correlation coefficient analysis.

	TPC	FRAP	DPPH	ABTS
TPC	1	0.962**	−0.995**	−0.937**
FRAP	0.962**	1	−0.958**	−0.912**
DPPH	−0.995**	−0.958**	1	0.937**
ABTS	−0.937**	−0.912**	0.937**	1

** Indicates significant correlation.

### 3.7 Antioxidant Capacity of Insect Oil

Many studies have confirmed that phenolic compounds possess exceptionally high antioxidant capacity, and TPC is often an essential indicator of a substance's antioxidant effectiveness, showing a positive correlation with antioxidant activity (Hassan and Swet Fan 2005; Kaur and Kapoor 2002; Liu et al. 2011; Muddathir et al. 2017; Zengin et al. 2015). The results of the present study are in agreement with the aforementioned findings, indicating that TPC of oil extracted from BSF larvae has strong correlations with ferric reducing ability, DPPH scavenging capacity, and ABTS scavenging ability. These findings are consistent with those of Muangrat and Pannasai (2024), Phongpradist et al. (2023), and Ravi et al. (2019).

The aforementioned findings also support the potential of oil extracted from BSF as a valuable antioxidant. This study found that larvae feeding substrate significantly influenced TPC and antioxidant capacity in extracted oil. Oil extracted from red‐quinoa‐fed larvae had the highest TPC, followed closely by oil extracted from rice‐bran‐fed larvae. Oils extracted from larvae reared on wheat bran or sorghum had significantly lower TPC values than did the aforementioned oils, with oil extracted from sorghum‐fed larvae exhibiting the lowest TPC. These results suggest that oil extracted from red‐quinoa‐fed larvae has the strongest antioxidant effect and that oil extracted from sorghum‐fed larvae has the weakest antioxidant effect. Because BSF larvae composition is influenced by feed, differences in antioxidant activity among larval oils may be attributed to variations in the food matrix. Studies have shown that byproducts of grain processing are rich in phenolic compounds (Fărcaș et al. 2021). Unprocessed red quinoa contains a husk with strong antioxidant properties, and its TPC can range from 21.15 mg GAE/g substrate (Kuo et al. 2021) to 35.53 mg GAE/g substrate (Li et al. 2020). The present study used whole‐husk red quinoa as feed, which might explain why the oil extracted from red‐quinoa‐fed larvae exhibited the strongest antioxidant effects. Similar to red quinoa, rice bran has a high antioxidant capacity, with its TPC ranging from 3.2 to 12.4 mg GAE/g substrate (Rao et al. 2010). The oil extracted from larvae reared on rice bran performed well in the DPPH scavenging tests, with its DPPH scavenging activity being slightly lower than that of Trolox as a standard reference (Phongpradist et al. 2023). Moreover, wheat bran has notable antioxidant properties, with its TPC ranging from 2.29 to 3.05 mg GAE/g substrate (Yu and Zhou 2005). Red wheat bran, which was used in the present study, exhibits better antioxidant effects than does white wheat bran, with the TPC of red wheat bran being up to 3.967 mg GAE/g substrate (Yu and Zhou 2005). The antioxidant effects of sorghum vary significantly with its variety (Dlamini et al. 2007; Shen et al. 2018). The variety of sorghum used in this study was not known. Nevertheless, research suggests that the color of sorghum can be used to determine its antioxidant capacity. In particular, brown sorghum has a weaker antioxidant effect than does sorghum of any other color, with TPC of brown sorghum ranging from 2.1 to 3.58 mg GAE/g substrate (Dykes et al. 2005; Rao et al. 2018). Brown sorghum was used in the present study, which might be one reason why the oil extracted from sorghum‐fed larvae exhibited the weakest antioxidant performance. Overall, the nutritional value of BSF larvae depends on the food matrix, and their antioxidant capacity is influenced by the composition of their feed. These findings confirm the close relationship between diet and insect body composition.

### 3.8 Comparative Analysis of Substrate Efficacy

A comparative assessment of the four substrates reveals a clear trade‐off between larval productivity and oil bioactivity. Sorghum‐fed larvae achieved the highest oil yield (23.96%), followed by wheat bran (22.61%), yet both produced oils with the lowest antioxidant capacity (TPC ≤ 52.84 ± 1.07 μg GAE/g). In contrast, red quinoa hulls produced oils with the highest TPC (79.25 ± 1.04 μg GAE/g), approximately 2.1‐fold higher than sorghum, and demonstrated the strongest radical scavenging activity across all three assays. However, red‐quinoa‐fed larvae were the smallest and yielded only 20.27% oil extraction. Rice bran emerged as the optimal compromise, achieving high antioxidant capacity (TPC = 76.30 ± 2.55 μg GAE/g) while maintaining moderate oil yield (17.42%). These findings establish a practical framework for substrate‐dependent optimization of BSF oil bioactivity. As emphasized by Siddiqui et al. (2025), enhancing both the bioconversion rate and end‐product quality is critical for sustainable BSF farming. Our results demonstrate that substrate selection directly modulates these parameters, enabling producers to prioritize either maximum yield (sorghum/wheat bran) or premium functional oil quality (red quinoa/rice bran) based on market requirements and end‐use applications.

### 3.9 Future Prospects and Limitations

Although this study underscores the potential of utilizing cereal byproducts to enhance the functional quality of BSF oil, several limitations and avenues for future research should be addressed. While the TPC was found to be strongly correlated with antioxidant bioactivity, the specific bioactive constituents responsible for these effects have yet to be fully identified. A detailed profiling of individual phenolic acids and fatty acid compositions by employing advanced analytical techniques such as GC–MS or HPLC would provide a more profound understanding of the nutrient transfer and bioaccumulation mechanisms from the substrate to the larvae (Mannawa Arachchige et al. 2025).

Furthermore, it is essential to acknowledge the inherent heterogeneity of agro‐industrial byproducts. The chemical composition of substrates like rice bran and red quinoa hulls can vary significantly depending on crop variety, cultivation conditions, and post‐harvest processing methods (Das et al. 2025; Huang et al. 2025). Such nutritional fluctuations, particularly regarding fiber content and micronutrient profiles, may influence the metabolic conversion efficiency of BSF larvae and the subsequent reproducibility of the oil's antioxidant properties (Opoku et al. 2023). For large‐scale industrialization, developing standardized substrate formulations or implementing stabilization pre‐treatments will be crucial to ensuring consistent quality and bioactivity in the extracted BSF oil (Siddiqui et al. 2024; Tariq et al. 2025).

## 4 Conclusion

In summary, the findings of this study indicate that the cereal byproduct used to feed BSF larvae influences their physiology and the functional quality of the oil extracted from them. The use of red quinoa feed maximizes TPC and radical scavenging activity but results in relatively small larvae and a low oil yield. By contrast, the use of sorghum and wheat bran feed enhances oil yield but decreases TPC. Rice bran emerges as a balanced feeding substrate, offering strong antioxidation ability coupled with moderate larval growth and oil yield. Therefore, rice bran represents the most practical feedstock for producing BSF oil with both economical and functional advantages. The findings of this study can serve as a reference for the sustainable valorization of cereal processing residues and for the optimization of insect‐based lipid production in nutraceutical, cosmeceutical, and other value‐added applications.

## Author Contributions

**Cheng‐You Chen:** data curation. **Yung‐Sheng Lin:** formal analysis, writing – original draft, writing – review and editing. **Cheng‐Kun He:** writing – original draft, writing – review and editing. **Yu‐Ting Zhu:** data curation. **Chin‐Tung Wu:** conceptualization, formal analysis, writing – original draft, writing – review and editing.

## Funding

This study was supported by the National Science and Technology Council of Taiwan (Grant No. NSTC113‐2622‐E‐224‐011).

## Conflicts of Interest

The authors declare no conflicts of interest.

## Data Availability

The data that support the findings of this study are available from the corresponding author upon reasonable request.
